# The efficacy of ketamine supplementation on pain management for knee arthroscopy: A meta-analysis of randomized controlled trials: Erratum

**DOI:** 10.1097/MD.0000000000016918

**Published:** 2019-08-16

In the article, “The efficacy of ketamine supplementation on pain management for knee arthroscopy: A meta-analysis of randomized controlled trials”,^[1]^ which appears in Volume 98, Issue 27 of *Medicine*, Dr. Huiqin Xue should also be listed as the corresponding author. The corresponding author information for both corresponding authors should be:

Correspondence: Teng Ma, No. 5 Xinhua Street, Huimin District, Huhhot, Inner Mongolia. Fundamental Department of Nursing School of Inner Mongolia Medical University, 010059 (e-mail: mateng820023@163.com). Huiqin Xue, Nursing School of Inner Mongolia Medical University, Inner Mongolia, China (xuehuiqin1977@163.com).

Drs. Yawen Shen and Huiqin Xue's affiliation should be Nursing Department, The Second Affiliated Hospital of Inner Mongolia Medical University, Inner Mongolia, China.

Affiliation a should appear as Department of Orthopedics and Traumatology, The Second Affiliated Hospital of Inner Mongolia Medical University, Inner Mongolia, China.
